# Complete Sequence, Genome Organization and Molecular Detection of Grapevine Line Pattern Virus, a New Putative Anulavirus Infecting Grapevine

**DOI:** 10.3390/v12060602

**Published:** 2020-05-31

**Authors:** Toufic Elbeaino, Levente Kontra, Emese Demian, Nikoletta Jaksa-Czotter, Amani Ben Slimen, Richard Fabian, Janos Lazar, Lucie Tamisier, Michele Digiaro, Sebastien Massart, Eva Varallyay

**Affiliations:** 1 Istituto Agronomico Mediterraneo di Bari, Via Ceglie 9, 70010 Valenzano, BA, Italy; elbeaino@iamb.it (T.E.); b.slimen.a@gmail.com (A.B.S.); digiaro@iamb.it (M.D.); 2 Agriculture Biotechnology Research Institute, National Research and Innovation Center, Szent-Gyorgyi A street4, 2100 Godollo, Hungary; kontra.levente@abc.naik.hu (L.K.); emese.demian@abc.naik.hu (E.D.); czotter.nikoletta@abc.naik.hu (N.J.-C.); richard6fabi@gmail.com (R.F.); 3 Research Institute for Viticulture and Enology, Experimental Station of Kecskemét, National Research and Innovation Center, Katona Jozsef street 5, 6000 Kecskemét, Hungary; lazar.janos@abc.naik.hu; 4 Plant Pathology Laboratory, TERRA-Gembloux Agro-Bio Tech, University of Liège, Passage des Déportés, 2, 5030 Gembloux, Belgium; lucie.tamisier@uliege.be (L.T.); sebastien.massart@uliege.be (S.M.)

**Keywords:** grapevine, virus, line pattern, anulavirus, small RNA, HTS

## Abstract

Grapevine line pattern virus (GLPV) was first described 30 years ago in Hungary. The lack of its genomic sequences and of an available antiserum made its detection impossible in other parts of the world. Three different high-throughput sequencing (HTS) protocols applied on a GLPV-infected vine allowed the construction of the full genome sequence of this virus. It includes three RNA segments, encoding four proteins: methyltransferase-helicase (1a), RNA-dependent RNA polymerase (2a), movement protein (3a) and coat protein (3b). The obtained sequences were used to design specific primers for its detection by RT-PCR and Northern blot hybridization, respectively. These diagnostic methods were used to test the presence of GLPV in graft-inoculated plants and in 220 grapevine accessions of different Mediterranean origins. The three RNAs-encoding proteins of GLPV shared a very high amino acid identity with those of hop yellow virus, a tentative member of the *Anulavirus* genus, leaving no doubt that both are two isolates of the same viral species. A circular RNA originating from the RNA2 was found, for which an alternative silencing suppressor role is hypothesized. Further investigation is needed to determine this possibility and also the host range and pathological significance of the virus.

## 1. Introduction

The number of grapevine-infecting viruses (over 80) has increased drastically in recent years [1], mainly due to high-throughput sequencing technology (HTS). Line pattern disease of grapevine, characterized by bright yellow discolorations of the leaves and rings of variable size, scatted spots or blotches or Norway maple leaf-like patterns, was first reported in Hungary in the eighties, on *cvs* Jubileum 75, Limberger and Irsai Oliver [2]. Infected grapevines showed the presence of polymorphic virus-like particles that could be mechanically transmitted onto a range of herbaceous hosts comprising species of the families *Chenopodiaceae*, *Solanaceae*, *Cucurbitaceae*, *Leguminosae*, *Amaranthaceae* and *Aizoaceae* [3]. Based on the particles’ morphology, this virus was thought as a possible member of the *Ilarvirus* genus in the family *Bromoviridae*, and the name grapevine line pattern virus (GLPV) was assigned to it. Although GLPV is included among the viruses that infect the grapevine ([4,5,6]), its hitherto unknown genomic sequence, together with the lacking serological assay, have both penalized its finding in grapevine in other parts of the world and its characterization. 

Since its first discovery, two other ilarviruses have been reported from *Vitis* spp.: grapevine angular mosaic virus (GAMV) in Greece [7], which has a particle morphology different from that of GLPV, and grapevine virus S (GVS) in the USA, for which only two partially sequenced RNAs (JX513898 and JX513899) are available in GenBank (M.E. Rott and M. Belton, unpublished), without any symptom description that would not allow its comparison to GLPV.

Serological tests have also excluded the probable correlation of GLPV with alfalfa mosaic virus (AMV), a species belonging to the genus *Alfamovirus* and an agent of the yellow mottle disease, with a multipartite genome and different particle shapes [3,4]. 

Raspberry bushy dwarf virus is another grapevine-infecting virus belonging to *Idaeovirus,* a genus with several common characteristics with ilarviruses (isometric particles, multipartite genome, seed and pollen transmission), whose presence has been found in line pattern-diseased vines in Slovenia, Serbia and Hungary [8,9,10]. In this species, the presence of a circular RNA originating from RNA2 was shown [11], which was also confirmed in another *Idaeovirus,* blackcurrant leaf chlorosis-associated virus, suggesting the possible coexistence in these species of linear and circular viral RNAs [12].

The family *Bromoviridae* is currently composed of six different viral genera (*Alfamovirus*, *Anulavirus*, *Bromovirus*, *Cucumovirus*, *Ilarvirus* and *Oleavirus*) characterized by spherical to bacilliform viral particles, with a multipartite genome, approximately 8 kb long, consisting of three linear, positive sense ssRNAs with 5′-terminal cap structures [11]. Each genomic RNA1 and -2 encodes a single large open reading frame (ORF) involved in the viral replicase. RNA3 contains two ORFs encoding the movement protein (MP, 3a protein) and the coat protein (CP, 3b protein), expressed from a sgRNA. Unlike other species of the family *Bromoviridae*, members of the genus *Anulavirus* are characterized by having an RNA3 slightly larger than RNA2 and a smaller 2a protein (78.9 kDa), and, differently to the genome of cucumoviruses and some ilarviruses, the protein 2b, encoding a viral silencing suppressor (VSR) of RNAi, is missing [13]. 

Currently, only two viral species are officially recognized as members of the genus *Anulavirus,* i.e., pelargonium zonate spot virus (PZSV), which is the type member [14,15] and Amazon lily mild mottle virus (ALiMMV) [16]. Another tentative anulavirus is cassava Ivorian bacilliform virus (CIBV), found in cassava plants in the north-west of the Ivory Coast [17].

In this paper, we describe the molecular features of GLPV, whose complete genome sequence was obtained through HTS and Sanger sequencing of RT-PCR amplicons, and which was found to be a putative member of the genus *Anulavirus*.

## 2. Materials and Methods 

### 2.1. Plant Materials

Since its original description in the eighties, GLPV has been maintained on two vines of an interspecific hybrid (Baco 22A) in a grapevine virus repository at Kecskemét-Katonatelep. In 2015, cuttings from one of the two Baco 22A vines were collected, sprouted, and RNA was extracted from their leaves, flowers, tendrils and shot tips using CTAB-based RNA purification [18]. Extracted RNAs were used in sRNA and RNA HTS, RT-PCR and Northern blotting assays. In 2018, canes of the second GLPV-infected Baco 22A vine were collected, sprouted and used for dsRNA HTS.

The presence of GLPV was investigated in 220 grapevine accessions of different Mediterranean origins. Total nucleic acids (TNA) for that assay were extracted from leaf vein tissues according to Foissac et al. [19].

### 2.2. Sample Preparation and Sequencing

For small RNA (sRNA) sequencing, RNA extracts originated from different organs were pooled and a sRNA fraction was purified from polyacrylamide gel. sRNA sequencing libraries were prepared from this purified fraction using TruSeq Small RNA Library Preparation Kit, following the manufacturers’ instructions (Illumina, San Diego, USA), with some modifications [20]. Samples were sequenced using a single index on a HiScan2000 by UD Genomed (Debrecen, Hungary) 50 bp, single end. Fastq files of the sequenced libraries were deposited at GEO database (accession number GSE148246). 

For ribodepleted RNA seq, RNA extracted from the shoot tip was used. The ribosomal RNA (rRNA) was removed using the Ribo-Zero™ Plant Leaf Kit (Illumina, San Diego, USA) and the library was prepared using the TruSeq Stranded Total RNA Library Prep Kit (Illumina, San Diego, USA). The samples were sequenced on the Illumina Nextseq 500 platform with paired sequencing of 2 × 150 nt at the GIGA facilities of Liège University (Liege, Belgium). Fastq files of the RNA seq can be accessed at SRA with the accession number PRJNA623589.

Tissues from leaves (20 gr) were used for viral dsRNA template extraction using the cellulose CC-41 (Sigma Aldrich, Milan, Italy) as described by Elbeaino et al. [21]. DsRNA were reverse-transcribed (cDNA) using a TruSeq RNA Sample Prep Kit v2 (Illumina, San Diego, USA) and the cDNA library was subjected to Illumina sequencing with a run of 50-bp single reads on a HiScan SQ apparatus (Illumina). 

### 2.3. Bioinformatics Analysis of the HTS

#### 2.3.1. Analysis of Small RNA Sequencing

The CLC Genomics Workbench (CLCbio, Aarhus, Denmark) was used for bioinformatics analysis. After trimming and quality control, reads were used for de novo assembly to build longer contigs (Table S1). The annotation of these contigs was performed using BLAST with the GenBank plant host virus reference collection (available at 03.07.2019). For the viruses which were represented by a contig, the reads were mapped to the reference genome and were counted with and without redundancy. The normalized redundant read count was calculated as reads/1 million sequenced reads. Based on this mapping, a consensus sequence was assembled and used to calculate the percent (%) coverage of the viral genomes (Table S2).

#### 2.3.2. Analysis of Ribodepleted RNA Sequencing

The reads were trimmed for adapters and sorted by sample using Basespace (Illumina) (Table S1). The Geneious prime 2019.0.3 software (Biomatters, New Zealand, https://www.geneious.com) was used for sequence analysis. A total of 9,503,900 paired reads (2 × 150 nt) were obtained. Paired reads merged using BBMerge and duplicated reads were eliminated by Dedup embedded in Geneious prime. Deduplicated reads were used for de novo assembly by SPAdes assembler software (version 3.10.0) using the default parameters of the Geneious plugin. The annotation of de novo contigs performed using both Geneious’s BLASTX and BLASTN modules was based on a custom database created from the NCBI’s downloaded viral RefSeq database (available at 03.07.2019). The deduplicated unique reads were mapped to the reference genomes of the obtained virus hits using the Geneious mapper (Table S2). 

#### 2.3.3. Analysis of dsRNA Sequencing

The raw reads obtained were filtered, reduced to unique reads and assembled de novo into larger contigs using Velvet software [22] (Table S1). Assembled contigs were then screened for sequence similarity using BLASTX (cutoff e-value 10^−6^) and BLASTP.

### 2.4. Completing the Full Genome Sequence of GLPV

Anulavirus-related sequences were reconstructed by aligning homologous contigs to sequences of four viruses (ALiMMV, PZSV, CIBV and HYV) using “Geneious Prime” software (www.geneious.com). The complete sequences of viral RNA segments of GLPV were rechecked by sequencing RT-PCR amplicons obtained from the use of sets of sense and antisense-specific primers designed on assembled genomic RNA sequences (Table S3). The 5′- and 3′-terminal sequences of viral RNAs were determined by the sequencing of RT-PCR clones obtained by 5′/3′ RACE-PCR (Roche Diagnostics, Milan, Italy). All PCR products were ligated into the StrataCloneTM PCR cloning vector pSC-A (Stratagene, USA) and sequenced. Sequences were aligned with CLUSTALX 1.8 [23]. BLASTX and BLASTP programs were used to search for sequence homologies. The hairpin-loop structures at 5′ and 3′ termini were predicted using MFOLD [24].

### 2.5. Phylogenetic Analysis of GLPV

Amino acid sequences of RdRP and CP genes of GLPV, and those of members of the six genera belonging to the family Bromoviridae, were retrieved from the GenBank and used in the phylogenetic trees that were generated by the “Neighbour-joining” method implemented in MEGA5.05 [25].

### 2.6. Validation of HTS with Independent Methods

#### 2.6.1. RT-PCR

From the Baco 22A vine, the cDNA was synthetized from individual vine extracts and also from pooled RNAs, which served as a base for the preparation of the sRNA library using a random primer of the RevertAid First Strand cDNA Synthesis Kit (Thermo Fisher Scientific, Waltham, MA, USA), according to the manufacturers’ instructions. The generated cDNA was used as template for PCR reactions using Q5 DNA Polymerase (New England Biolabs) and primers, which were designed based on the sequenced reads (Table S3). PCR products were analyzed by agarose gel electrophoresis and by Sanger sequencing after cloning into pJET1.2 vector (Thermo Fisher Scientific, USA). RNAs, extracted from vines of different Mediterranean origins, were randomly reverse-transcribed using the *M-MLV* reverse-transcriptase enzyme, according to the manufacturers’ instructions (Thermo Fisher Life Technologies, USA), and used as a template in RT-PCR using GLPV-specific primers designed on the CP gene sequence (Table S3).

#### 2.6.2. Northern Blot Hybridization

For Northern blot analyses, 3 µg of total RNA were separated on a denaturing-formaldehyde 1.5% agarose gel and blotted to Amersham Hybond-NX membrane (GE Healthcare, Chicago, IL, USA) by the capillary method using 20× SSC (3 M NaCl and 0.3 M Na-citrate; pH 7.0). Hybridization was carried out at 65 °C in Church buffer (0.5 M Sodium Phosphate buffer, pH 7.2 containing 1% BSA, 1 mM EDTA, 7% SDS) overnight with the appropriate radioactive probe, washed for 5 min in 2× SSC, 0.1% SDS and for 15 min in 0.5× SSC, 0.1% SDS at hybridization temperature and exposed to an X-ray film. Virus-specific, P^32^-labelled DNA probes were prepared using the DecaLabel DNA Labelling Kit (Thermo Fischer Scientific). PCR-amplified and purified products of the cloned regions from all three RNAs were used as a template. Two membranes were prepared and hybridized with RNA2 and RNA3-specific probes. After exposure, the membrane hybridized with the RNA2-specific probe was stripped and rehybridized with the RNA1-specific probe.

### 2.7. Identification and Validation of a GLPV-Derived Circular RNA

The presence and the nature of a circular RNA (derived from the RNA2), identified in the HTS-generated contigs of the analyzed sequences, were ascertained by RT-PCR assay using two pairs of sense and antisense primers designed at a common position on the circular RNA sequence (Table S3), which enables DNA synthesis in both directions. The cDNA synthesized from RNA extracted from the inflorescence was used as a template for the PCR reaction. The PCR amplicon was cloned and Sanger-sequenced in order to determine its GLPV-origin. Its sequence was deposited into NCBI GenBank (accession number: MT358428).

Secondary structure prediction of the circular RNA was done using MFOLD [24]. 

### 2.8. Graft Transmissibility of GLPV

In 2016, the buds of GLPV-infected Baco 22A accession were grafted by the chip budding method onto four varieties, i.e., Harslevelu, Jubileum 75, Irsai Oliver and Kekfrankos, with three repetitions for each variety. In the following years (2017–2019), all grafted plants were tested for the presence of GLPV by Northern blot (2017 and 2018) and RT-PCR (2019) assays.

## 3. Results and Discussion

### 3.1. An Anulavirus is Detected in the GLPV Symptomatic Vine 

**Figure 1:**
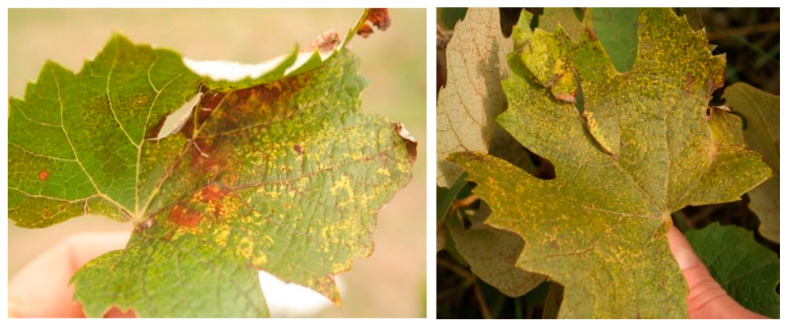
Symptoms of yellow ringspots on leaves of the Baco 22A rootstock in 2017.

Two Baco 22A vines, used for maintaining GLPV, were sampled and used for HTS. They showed spots, and yellow circle-like motifs, which were described as typical symptoms in the chronic phase of GLPV infection (Figure 1). 

The HTS-library analyses of sRNA and ribodepleted RNA seq revealed the presence of viral sequences belonging to grapevine Pinot gris virus (GPGV), grapevine red globe virus (GRGV), grapevine rupestris stem pitting-associated virus (GRSPaV) and of viroidal sequences, i.e., hop stunt viroid (HSVd) and grapevine yellow speckle viroid 1 (GYSPVd-1) (see Table S1 for data on basic statistics and Table S2 for the detailed results), but these data have not been further investigated and discussed in this paper. In addition, significant hits with three RNAs (RNA1 of 3107 nt, RNA2 of 2446 nt and RNA3 of 2513 nt) composing the genomes of ALiMMV, CIBV and PZSV members of the same genus Anulavirus (Table S2) were also obtained.

From the de novo assembled contigs of the dsRNA HTS (see Table 1 for basic statistics), 792 contigs were identified with similarity to genes encoded by different grapevine-infecting viruses, i.e., closteroviruses (grapevine leafroll-associated virus 1, 2 and 3); betaflexiviruses (GRSPaV) and maculaviruses (grapevine fleck virus). This result is different from those obtained with sRNA and RNA seq, due to the use of a different grapevine source. Another 361 contigs resembled the genes of anulaviruses, while the remaining contigs did not yield any sequence identity with known viruses reported in the GenBank. Although a very limited number of RNA contigs spanning short stretches of sequences were obtained from the analyzed HTS-cDNA library, all indicated in BLAST search that GLPV shares a high genetic identity with ALiMMV, reconfirming its previous tentative taxonomic classification [26].

The identified contigs were taken as a proof that GLPV belongs to the Anulavirus genus, and allowed us to design primers for amplifying longer regions of each RNA.

### 3.2. Completion of Three Genomic RNA Sequences of GLPV

**Figure 2:**
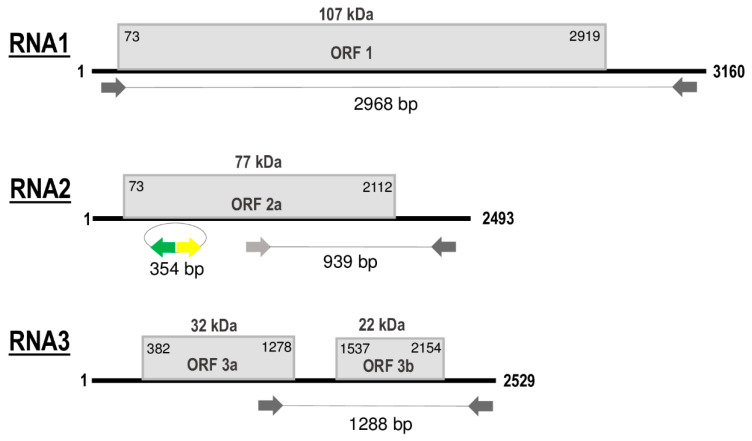
Schematic representation of genomic RNA segments of grapevine line pattern virus (GLPV). open reading frames (ORFs) are indicated as boxes on each segment. Initiation and termination positions of the coding regions are indicated above each box. Expression product of each RNA [1a (Methyltransferase\Helicase, MTR-HEL); 2a (RNA-dependent RNA Polymerase, RdRP), 3a (Movement protein, MP) and 3b (Coat protein, CP)] is reported inside the boxes. The molecular weight predicted for each protein is reported above boxes. Amplified parts which were used in the RT-PCR and Northern validation are also indicated. Figure not drawn to scale.

Sequences obtained from the HTS library, Sanger-sequencing of RT-PCR amplicons that covered all genomic segments, and those from 5′ and 3′ RACE, showed that the genome of GLPV is composed of three ssRNA segments (RNA1 -MT319109, RNA2 - MT319110, RNA3 - MT319111) (Figure 2).

RNA1, 3160 nt in length, contains a single open reading frame (ORF1) of 2847 nt that encodes a polypeptide of 948 amino acids (aa), with a predicted molecular weight (Mw) of 107 kDa, denoted as domain 1a (Methyltransferase\Helicase, MTR\HEL). ORF1 initiates with a start codon (AUG) at nt position 73 and terminates with an ochre stop codon (UAA) at position 2919. The untranslated regions (UTR) at both 5′ and 3′ terminal sequences are 73 and 241 nt in size, respectively (Figure 2). The 1a protein showed to share the highest aa identities (ranging from 47% to 82%) with the comparable protein of members (PZSV, ALiMMV) and tentative member (CIBV) of the genus *Anulavirus* (Table 1). BLAST search of the fully sequenced GLPV genome revealed a very high sequence identity (99%) with hop yellow virus (HYV), another tentative member of the *Anulavirus* genus not yet officially published and recognised, but which is fully sequenced. Two isolates from China, HYV-China (accession numbers: MG727388, MG727389 and MG727390) (C. Yu, unpublished) and HYV-Nb (MN520240, MN520241, MN520242) (Y. Lu, unpublished), are reported in GenBank, infecting hop (*Humulus lupulus* L.) and Thunberg fritillary (*Fritillaria thunbergii* Miq.), respectively.

RNA2 of GLPV, 2493 nt in length, contains ORF2 of 2040 nt, whose translation starts at nt position 73 with an AUG and ends at position 2112 with an UAA stop codon (Figure 2). The sequence length of the 5′UTR is similar to that of RNA1 (73 nt), whereas the 3′UTR is 381 nt long. ORF2 encodes a polypeptide of 679 aa, with a predicted Mw of 77 kDa, denoted as domain 2a (RNA-dependent RNA Polymerase, RdRP). BLAST analysis showed that the RdRP of GLPV shares aa identities of 92%–99%, 74%, 71% and 48%, with the comparable protein encoded by RNA2 of HYV (NB and China strains), CIBV, ALiMMV and PZSV, respectively (Table 1).

RNA3, 2529 nt in length, contains two ORFs that encode two proteins, denoted 3a (movement protein, MP) and 3b (coat protein, CP). The translation of the protein 3a initiates at nt position 382 and terminates at position 1278; the encoded protein of 298 aa has a predicted Mw of 32 kDa. The protein 3b starts at nt position 1537 and terminates at position 2154, encompassing 205 aa with a predicted Mw of 22 kDa. Similarly to GLPV RNA1- and RNA2-encoded proteins, the amino acid identity matrix of RNA3 showed again that the MP (RNA3a) and the CP (RNA3b) of GLPV shares the highest identity with their homologues of anulaviruses, and in particular with the two isolates of HYV (97% and 95–98%, respectively) and the lowest with PZSV (45% and 28%) (Table 1). The 3′UTR of RNA3 is 375 nt in length (Figure 2). The sequence alignment of the 5′ UTR showed that these regions are not only very similar to each other, but are extremely well conserved within the anulaviruses (Figure S1). 3′UTR of all RNA1-3 showed the same type of conservation within the genus (Figure S2).

### 3.3. Phylogenetic Analysis of GLPV

**Figure 3:**
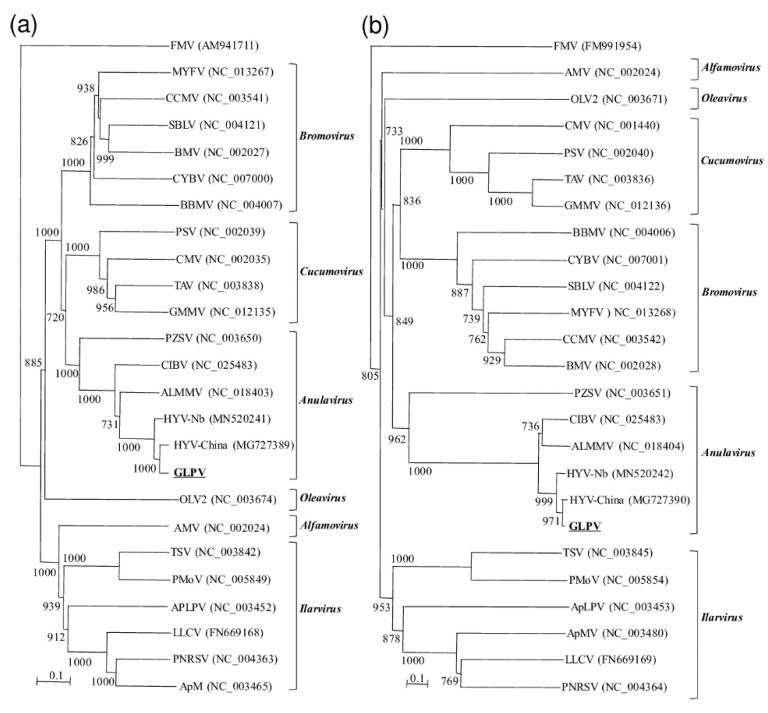
Phylogenetic trees, generated by the “Neighbour-joining” method, constructed based on aa sequences of the (**a**) RdRP and the (**b**) CP of GLPV and members of six genera belonging to the family Bromoviridae: Alfamovirus [alfa mosaic virus, (AMV)], Anulavirus [Amazon Lily mild mottle virus (ALMMV), pelargonium zonate sport virus (PZSV), and the two putative members: hop yellow virus (HYV, isolates China and Nb) and cassava Ivorian bacilliform virus (CIBV)], Bromovirus [broad bean mottle virus (BBMV), brome mosaic virus (BMV), cassia yellow blotch virus (CYBV), cowpea chlorotic mottle virus (CCMV), melandrium yellow fleck virus (MYFV), spring beauty latent virus (SBLV)], Cucumovirus [cucumber mosaic virus (CMV), Ggyfeather mild mottle virus (GMMV), peanut stunt virus (PSV), tomato aspermy virus (TAV)], Ilarvirus [apple mosaic virus (ApMV), prunus necrotic ring spot virus (PNRSV), lilac leaf chlorosis virus (LLCV), apple line pattern virus (ApLPV), pepper mottle virus (PMoV), tobacco streak virus (TSV)] and Oleavirus [olive latent virus 2 (OLV2)]. Fig mosaic emaravirus (FMV) was used to root the trees. Accessions numbers of viruses used are reported within brackets. Numbers on branches indicate percentage of support out of 1000 bootstrap replications. Bootstrap values above 70% are shown. Scale bar represents 0.1 aa substitutions per site.

The phylogenetic trees constructed based on the amino acid sequences of 2a and 3b also confirmed the belongingness of GLPV to the genus *Anulavirus*, allocating it in one cluster together with the anulaviruses and close to HYV (Figure 3).

In general, sequences of the three viral RNAs of GLPV shared at the nucleotide and amino acid levels an overall identity of 90–97% and 92–99% with those of HYV, suggesting that these two viruses are probably not two different species, but only different strains of the same virus. This result was also supported by the phylogenetic analysis, which allocated GLPV in a cluster with anulaviruses, close to the HYV isolates (China and Nb) reported in the GenBank (Figure 3).

### 3.4. RT-PCR and Northern Hybridization Confirmed the Presence of the Anulavirus

In RT-PCR, all primers designed on nucleotide sequences of RNA1, 2 and 3 showed to be specific and efficient to detect GLPV in the Baco 22A accession (Figure S3).

**Figure 4:**
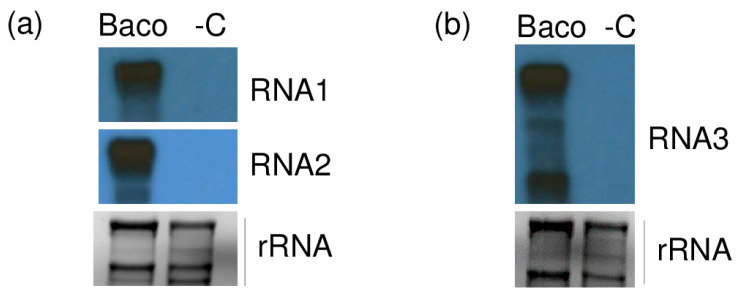
Validation of the presence of GLPV in a diseased vine by Northern blot. X-ray films exposed after hybridizations using (**a**) RNA1 and RNA2, (**b**) RNA3 radioactively labelled virus-specific riboprobes. “Baco” marks the RNA from the GLPV-infected vine, while “-C” the RNA from a non-infected vine. EtBr-stained agarose gels before blotting served as a loading control.

When used in Northern blot, all three riboprobes were able to hybridize their homologue of RNA1, 2 and 3 and sgRNA3 of GLPV, confirming the presence of the GLPV RNA segments only in Baco 22A accession but not in healthy plant material (Figure 4).

### 3.5. Identification of a Circular RNA2-Derivate Present in GLPV-Infected Plants

**Figure 5:**
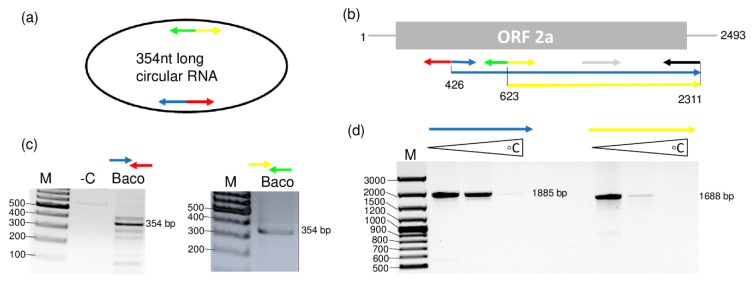
Validation of the circular RNA presence in the GLPV-infected grapevine. Strategies used to amplify the complete sequence of the (**a**) circular RNA (derived from RNA2) and (**b**) of the partial sequence of RNA2 of GLPV, using sense and antisense primers, both designed on sequences of RNA2, indicated by multi-coloured arrows. RT-PCR amplifications using (**c**) circular RNA-derivate and (**d**) RNA2-specific primers. M: GeneRuler 100 bp Plus DNA ladder (Thermo Fisher Scientific). Triangles show RT-PCR amplicons produced at different annealing temperatures (50–56–64 °C).

After the full genome sequence completion of GLPV, the HTS-contigs were again mapped to the GLPV genome in order to check for the presence of additional GLPV isolates with sequence variations within the library. All GLPV-contigs mapped were identical, with the exception of a contig of 167 nt long that shared high sequence identity with RNA2 of GLPV. When further analyzed for its secondary structure, this sequence showed to encompass a circular RNA conformity different from the homologue region of RNA2 of GLPV. To verify that this finding was not an artefact of the assembler and that this circular RNA really exists in the Baco 22A vine, sense and antisense primers (Figure 5a,b), both initiating from a common position on this sequence, were designed (Table S3). RT-PCR showed amplifications only in the Baco 22A vine and not in GLPV-free vines, with an amplicon that after cloning and sequencing showed to be 354 bp long (Figure S3 and Figure 5c).

The circular conformity of this RNA2-derivate sequence was proven through two different sets of primers that allow its amplification at two different starting points (Figure 5a,b) enabling the amplification of the whole RNA, but only in case it is circular (Figure 5a). The Sanger sequencing of the amplified products showed that they are identical to each other and this circular RNA shared 96% of nt identity and spanned a region between nt 349 and nt 703 on RNA2 of GLPV. The sequence was deposited in the GenBank under the accession number MT358428. This sequence similarity was also proven using a combination of circular RNA-specific sense primers and RNA2-specific antisense primers in an RT-PCR reaction that amplified products with the expected sizes (Figure 5d).

### 3.6. GLPV Induces Plant RNA Responses

**Figure 6:**
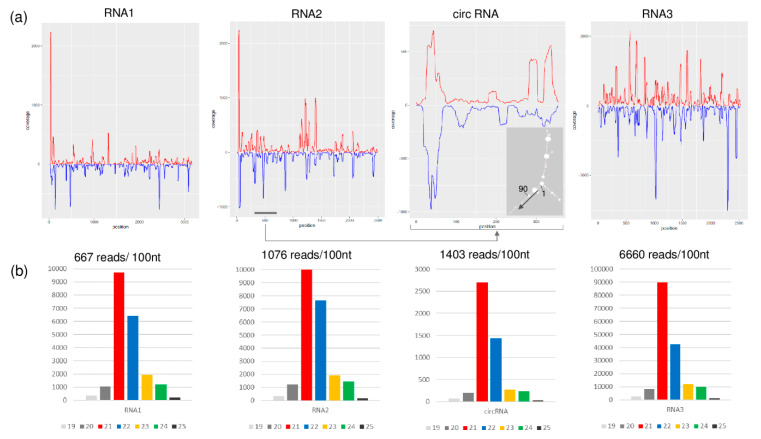
Profiles of GLPV-derived sRNAs. (**a**) distribution of the GLPV-derived sRNAs on the RNA1, RNA2, circular RNA and RNA3. Sense and antisense sequence orientations are indicated by red and blue colours, respectively. The small panel shows the secondary structure of the circular RNA (start position and direction are indicated). (**b**) Size distribution of GLPV-derived sRNAs based on their origin.

The availability of the full genome sequence of GLPV allowed us to study the presence and features of GLPV-derived sRNAs, which are the hallmarks of the plant’s immune response, indicating that a virus is actively replicating in them. SRNAs in both directions were generated by all three RNAs of GLPV in high number: 21.091; 26.828 and 168.456 reads (counted with redundancy) with no mismatch (Figure 6a). Their size was predominantly 21–22 nt, which is characteristic for the sRNAs produced by the Dicer-like proteins 2 and 4 (DCL2 and DCL4) during the antiviral silencing activity of the host (Figure 6b).

**Figure 7:**
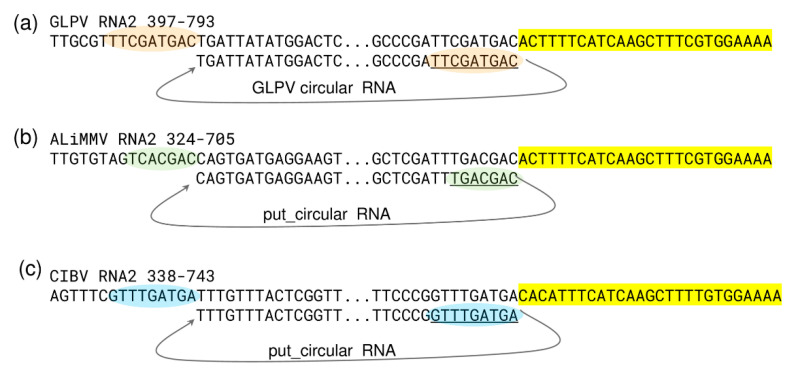
Nucleotide sequence analysis of the site of origin of the circular RNAs in anulaviruses. Nucleotides sequence alignments showing conserved regions (antiparallel) found at initiation and termination sites of the circular RNA in (**a**) GLPV (orange) and predicted in (**b**) ALiMMV (green) and in (**c**) CIBV (blue), together with conserved nucleotide stretches flanking these sites in the RdRP (highlighted in yellow).

As expected, most of the sRNA reads were derived from the structural RNA3 (6600 reads/1000 nt), which can be explained by the strong antiviral silencing-inducing activity of the abundant coat protein coding sgRNA. Several sRNA reads (1403 reads/1000 nt) were also generated from the circular RNA, as confirmed by the prediction analysis of their secondary structure, according to which most of them were produced by regions with extreme stem-loop structures (Figure 6a), further confirming the active replication of the circular RNA. Further analyses conducted on GLPV RNA2 sequence, regarding the possible biogenesis site of the circular RNA, revealed a sequence repetition of nine nucleotides before the initiation and the termination sites (Figure 7a). Similar sequence repetitions were also found on RNA2 of ALiMMV and CIBV immediately before a conservative motif in the RdRP coding region (Figure 7b,c), suggesting the possible presence of circular RNAs also in those viruses.

Anulaviruses do not encode a VSR on their RNA2; however, the presence of the circular RNA originating from RNA2 might suggest a possible alternative mechanism to block the host’s defence reaction. The sRNAs analysis showed that reads produced by the circular RNAs (1403 read/100 nt) were more abundant than those produced by any other part of the RNA2 (1076 read/100 nt) (Figure 7). It is possible that these sRNAs originated by circular RNAs, loaded into the host antiviral RNA-induced silencing complex (RISC), may interfere with the loading of other sRNAs generated by other parts of the genome. The loaded RISC could target the circular RNA according to its loaded siRNA and could give the other viral RNA a chance to escape the RISC activity and ensure the integrity of its structure. This finding could suggest that it is possible that in the absence of an efficient VSR, the anulaviruses may develop an alternative strategy to block the host RNAi, i.e., instead of a protein they code and replicate a circular RNA. Further investigations are, however, needed to confirm this theory.

### 3.7. Investigation of GLPV in Different Grapevine Organs and in Grafted Vines

The Northern blot assay, used to test the virus distribution in different organs of the plant, showed that GLPV was present in all organs examined (leaves, shoot tips, inflorescences and tendrils), but only from those of forced cuttings (Figure S4). Tests carried out on the grafted vines for the presence of GLPV using Northern blot or RT-PCR in three subsequent years (2017–2019) revealed that, although positive, virus transmission occurred at a low rate (Figures S5 and S6).

### 3.8. GLPV is not Present in Grapevine Accessions Collected from the Mediterranean

In an attempt to search for GLPV in grapevine accessions from different Mediterranean countries (Algeria, Albania, Greece, Italy, Lebanon, Malta, Palestine, Syria, Tunisia and Turkey), 220 grapevine vines, maintained under screenhouses in the premises of the Istituto Agronomico Mediterraneo of Bari, CIHEAM-IAMB (Italy), were assayed by RT-PCR using GLPV-specific primers designed on the sequence of the CP gene (Table S3). In RT-PCR, the positive controls yielded the expected amplicon of 430 bp in size, whereas all the other tested vines were negative, thus excluding a possible origin of GLPV in the Mediterranean region.

## 4. Conclusions

In this work we have determined the whole genome sequence of GLPV, almost 30 years after its first discovery. The characteristics of its genome (i.e., the tripartite genome, the presence of a smaller 2a protein, the absence of the 2b protein encoding a VSR, a slightly larger RNA3 than RNA2) and the high nucleotide identity with the genome of other anulaviruses, confirmed that GLPV is a putative member of the genus *Anulavirus*. Accordingly, it would be the fourth species of this genus, which currently comprises only two officially recognized members: PZSV and ALiMMV, and a tentative member: CIBV. The molecular information now gained on GLPV’s genome, together with those acquired in the eighties, i.e., disease characterization, shape of virus particle (polymorphic), possible member of the family *Bromoviridae* (confirmed in this study) and its ancient nomination as among the viruses that infect the grapevine [4], are all elements that legitimate its final classification and nomination in the ICTV as grapevine line pattern virus.

The molecular analysis conducted on its genome revealed and leaves no doubt that GLPV and HYV are two isolates of the same viral species. The sequences of two isolates of HYV, detected from hop and Thunberg fritillary plants, are reported in GenBank, but, apart from the genome sequence, no other information is currently available for them. Further investigation is needed to determine whether they infect the same plant hosts in nature or by artificial inoculation, how they are transmitted in nature, what their geographical distribution is, in order to conclude whether HYV should be considered as a distinct strain of GLPV or simply a GLPV isolate.

The absence of GLPV in several vines of Mediterranean origin seems to exclude its possible origin from that area and support the hypothesis that it may originate from areas with temperate/continental climates. This hypothesis seems to be substantiated by the detection of HYV (a GLPV isolate/strain) in China from hops, another typical crop of northern countries. However, further investigation is needed to confirm it.

Furthermore, a circular RNA originating from the RNA2 was identified. The possible biogenesis of a circular RNA from a linear viral genome was long unknown, until it was first reported in viruses of genus *Idaeovirus*, that are also characterized—like anulaviruses—by a multipartite genome. The cRNA of RNA2 origin found in our study might also recall defective RNA or DNA associated with the geminiviruses and multipartite RNA viruses [27,28], which could be involved in enhancing and/or suppressing host response to the virus infection. In the absence of the 2b protein-encoding gene in the RNA2 of anulaviruses, one can speculate that these circular RNAs may have the ability to alter the host RNAi and help the virus to survive. In theory, this strategy could work and may explain the successful survival of anulaviruses and of several idaeoviruses. However, in order to state that this mechanism really exists, the presence of circular RNAs of viral origin in infected plants and the presence and loading of sRNAs derived from circular RNAs into the host RISC should be further investigated and deepened in future studies.

## Figures and Tables

**Table 1:** Pairwise nucleotide and amino acid sequence identity (in %) of genomic RNAs of GLPV with corresponding regions of closely related members (and tentative members*) of the genus Anulavirus. Values for amino acids are shadowed. Underlined bold numbers indicate the highest identity found between GLPV sequences and those of other anulaviruses.

Virus	NCBI Reference	RNA1 (1a)					
		GLPV	ALiMMV	PZSV	HYV-Nb *	HYV-China *	CIBV *
**GLPV**	**MG727388**		82	47	97	**99**	74
**ALiMMV**	**AB724113.1**	76		45	82	82	74
**PZSV**	**KF790760.3**	50	50		47	47	46
HYV-Nb *	**MN520240**	**97**	77	50		96	74
HYV-China *	**MG727388**	92	76	50	92		74
CIBV *	**KF742519**	70	70	50	70	70	
		**RNA2 (2a)**					
**GLPV**	**MG727389**		71	48	92	**99**	74
**ALiMMV**	**AB724114.1**	69		47	70	72	67
**PZSV**	**LC178560.1**	51	46		46	48	49
HYV-Nb *	**MN520241.1**	90	69	50		92	71
HYV-China *	**MG727389**	**97**	69	51	90		74
CIBV *	**KF742520.1**	69	63	50	66	69	
		**RNA3 (3a)**					
**GLPV**	**MG727390**		86	45	97	**97**	80
**ALiMMV**	**AB724115.1**	81		46	88	88	84
**PZSV**	**KF790762.4**	49	49		45	45	47
HYV-Nb *	**MN520242.1**	94	80	49		99	81
HYV-China *	**MG727390.1**	**96**	81	49	96		81
CIBV *	**KF742521.1**	75	77	51	76	76	
		**RNA3 (3b)**					
**GLPV**	**MG727390**		85	28	95	**98**	83
**ALiMMV**	**AB724115.1**	81		28	87	86	86
**PZSV**	**KF790762.4**	43	41		28	28	30
HYV-Nb *	**MN520242.1**	95	81	42		95	84
HYV-China *	**MG727390.1**	**97**	83	43	94		84
CIBV *	**KF742521.1**	80	82	43	80	81	

## Supplementary Materials

The following are available online at https://www.mdpi.com/1999-4915/12/6/602/s1, Figure S1: Sequence alignment and secondary structure prediction of 5′UTR of RNA1 and RNA2 of GLPV and other anulaviruses. Figure S2: Sequence alignment and secondary structure prediction of 3′UTR of RNA1, RNA2 and RNA3 of GLPV and other anulaviruses. FigureS3: RT-PCR amplification of the GLPV. Figure S4: Investigation of the presence of GLPV in different organs and in two grafted vines by Northern blot. Figure S5: Testing GLPV transmission in different grapevine varieties after 2 years of grafting. Figure S6: Testing GLPV transmission in different grapevine varieties after 3 years of grafting. Table S1: Initial statistics of the different HTSs. Table S2: Results of the bioinformatics analysis of sRNA seq and RNAseq. Table S3: Sequences of the used primers for (a) RT-PCR and (b) for Sanger sequencing.
