# Supplementary material for: Complete Sequence, Genome Organization and Molecular Detection of Grapevine Line Pattern Virus, a New Putative Anulavirus Infecting Grapevine
Source: Viruses. 2020 May 31;12(6):602. doi: 10.3390/v12060602 (PMC7354484; doi:10.3390/v12060602)
Supplement: Supplementary file 1 [file viruses-12-00602-s001.zip › S1 Elbeaino et al_GLPV_Viruses_ve.docx]

Supporting S1 to

Complete sequence, genome organization and molecular detection of Grapevine line pattern virus, a new putative Anulavirus infecting grapevine.

Toufic Elbeaino^1x^, Levente Kontra^2x^, Emese Demian^2^, Nikoletta Jaksa-Czotter^2^, Amani Ben Slimen^1^, Richard Fabian^2^, Janos Lazar^3^, Lucie Tamisier^4^, Michele Digiaro^1^, Sebastien Massart^4^, Eva Varallyay^2^*

^1^Istituto Agronomico Mediterraneo di Bari, Via Ceglie 9, 70010 Valenzano, (BA), Italy. elbeaino@iamb.; [b.slimen.a@gmail.com](mailto:b.slimen.a@gmail.com); [digiaro@iamb.it](mailto:digiaro@iamb.it)

^2^Agriculture Biotechnology Research Institute, National Research and Innovation Center, Szent-Gyorgyi A street4, 2100, Godollo, Hungary. kontra.levente[@abc.naik.hu](mailto:@abc.naik.hu); [emese.demian@abc.naik.hu](mailto:emese.demian@abc.naik.hu); [czotter.nikoletta@abc.naik.hu](mailto:czotter.nikoletta@abc.naik.hu); [richard6fabi@gmail.com](javascript:void(window.open('/imp/dynamic.php?page=compose&to=richard6fabi%40gmail.com&popup=1','','width=820,height=610,status=1,scrollbars=yes,resizable=yes')))

^3^Research Institute for Viticulture and Enology, Experimental Station of Kecskemét, National Research and Innovation Center, Kecskemét, Hungary. [lazar.janos@abc.naik.hu](mailto:lazar.janos@abc.naik.hu)

^4^Plant Pathology Laboratory, TERRA-Gembloux Agro-Bio Tech, University of Liège, Passage des Déportés, 2, 5030 Gembloux, Belgium. lucie.tamisier@uliege.be; [sebastien.massart@uliege.be](mailto:sebastien.massart@uliege.be)

^x^These authors participated equally in the work

*****Correspondence: [varallyay.eva@abc.naik.hu](mailto:varallyay.eva@abc.naik.hu)


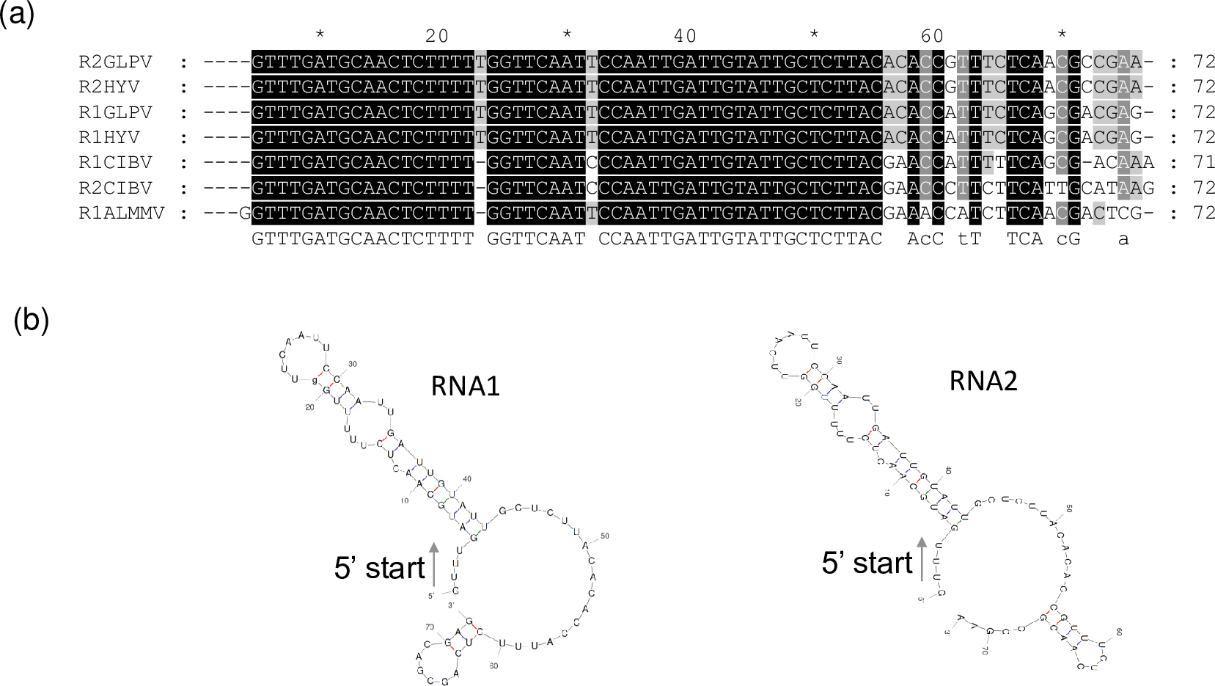


**Figure S1**. Sequence alignment and secondary structure prediction of 5’UTR of RNA1 and RNA2 of GLPV and other anulaviruses. (a) multiple alignment was prepared using CLUSTAL Omega, while (b) secondary structure prediction was prepared with MFOLD.


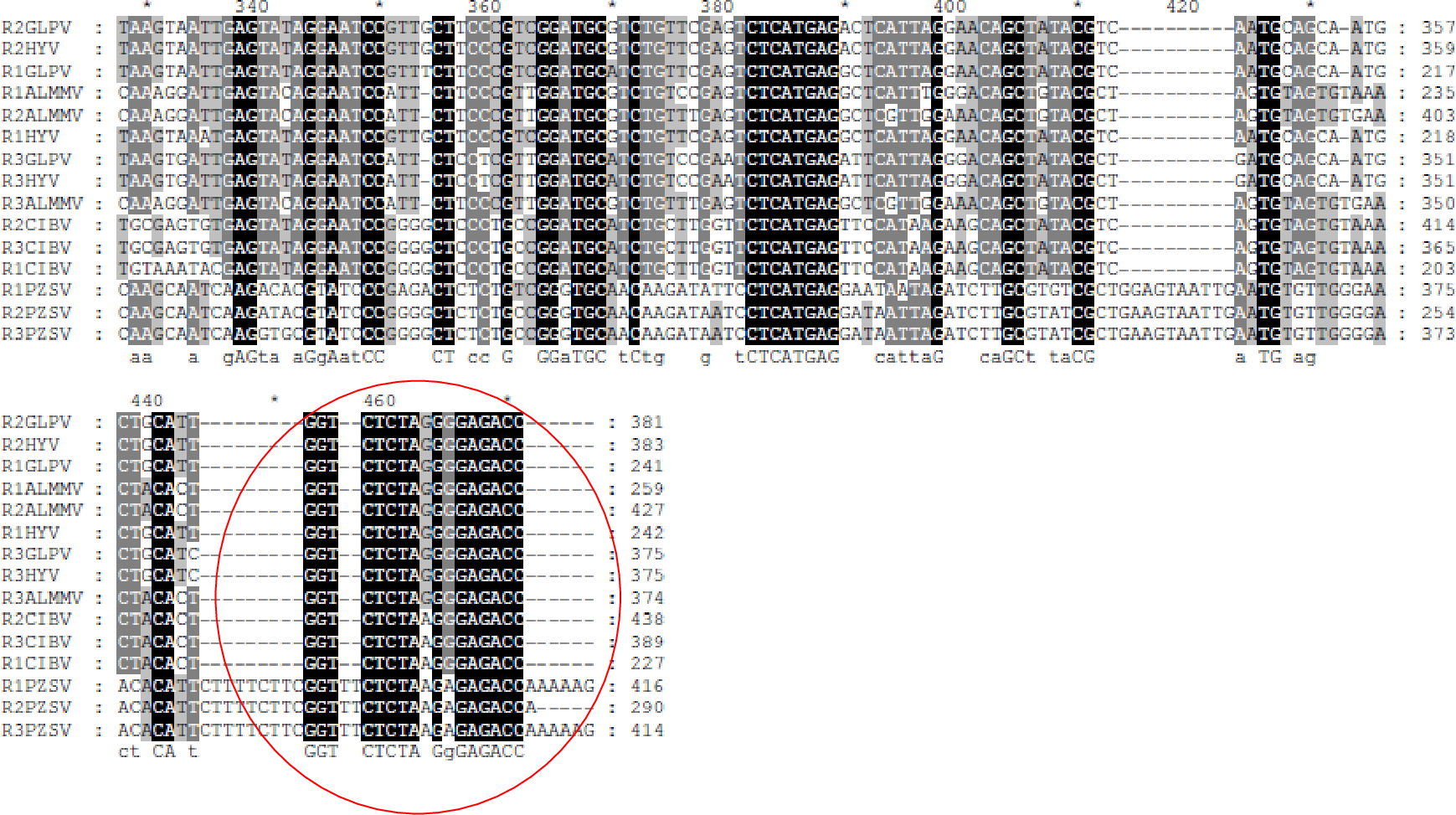


**Figure S2**. Sequence alignment and secondary structure prediction of 3’UTR of RNA1, RNA2 and RNA3 of GLPV and other anulaviruses.


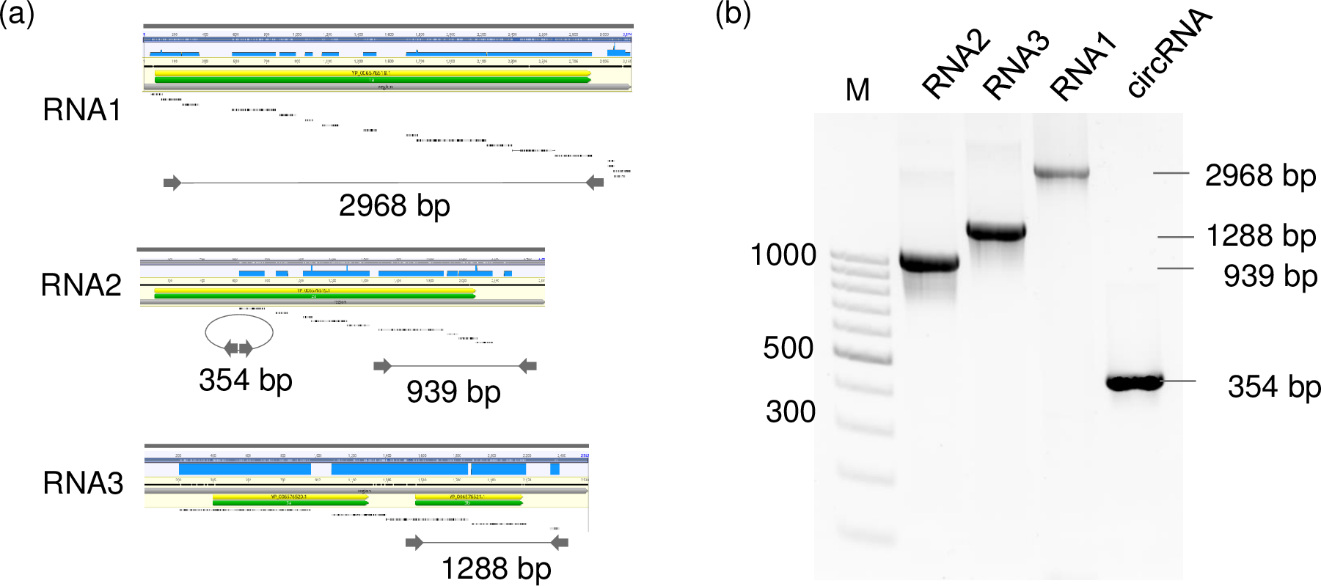


**Figure S3.** RT-PCR amplification of the GLPV. (a) position of the primers designed to each RNA segment of GLPV (b) RT-PCR amplifications obtained from the use of four couples of primer pairs specific to RNA1, RNA2, RNA3 and to the circular RNA (derived from RNA2). M: GeneRuler 100bp DNA ladder (Thermo Fisher Scientific, USA).


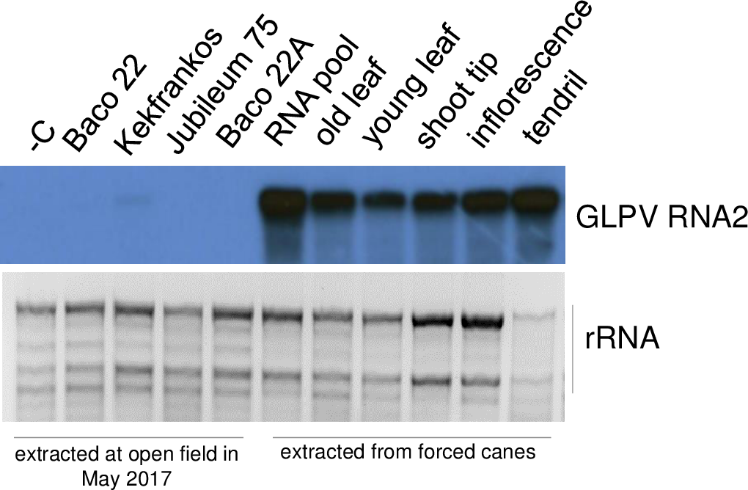


**Figure S4** Investigation of the presence of GLPV in different organs and in two grafts by Northern blot. RNA was extracted from different organs of the GLPV infected Baco 22A and from a non-infected grapevine (-C) as a negative control, separated and blotted into nitrocellulose membrane. The membrane was hybridized with virus specific radioactively labelled probes, using cloned, RNA1, RNA2, RNA3 and circular RNA specific fragments as templates. EtBr stained gels, highlighting the rRNA served as a loading control.


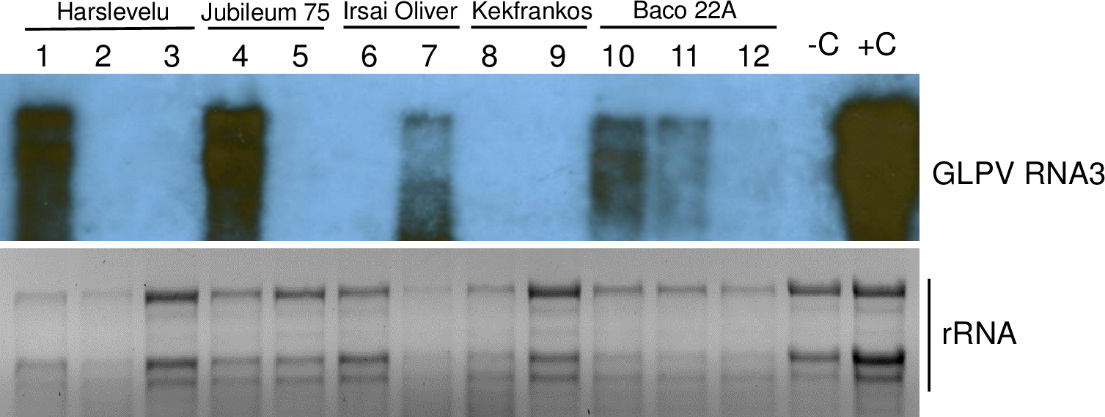


**Figure S5**. Testing GLPV transmission in different varieties after 2 years of grafting. Northern blot hybridization using radioactively labelled GLPV RNA3 specific probe were carried out to test the presence of the virus in different varieties. 1-3 Harslevelu, 4-5 Jubileum 75, 6-7 Irsai Oliver, 8-9 Kékfrankos, 10-12 Baco22, -C GLPV not infected Kadarka, +C RNA extract from Baco 22A forced canes. EtBr stained gels, highlighting the rRNA served as a loading control.





**Figure S6**. Testing GLPV transmission in different varieties after 3 years of grafting. RT-PCR using GLPV RNA3 specific primers were used to test the presence of the virus. 1-2 Harslevelu, 3-4 Jubileum 75, 5-6 Irsai Oliver, 7-8 Baco22, 9-21 GLPV not infected plants, -C GLPV not infected Kadarka, +C RNA extract from Baco 22A forced cutting.
